# Recent Advances in Strategies to Combat Bacterial Drug Resistance: Antimicrobial Materials and Drug Delivery Systems

**DOI:** 10.3390/pharmaceutics15041188

**Published:** 2023-04-07

**Authors:** Jiaxin Yao, Pengfei Zou, Yanan Cui, Liangzhu Quan, Chunsheng Gao, Zhiping Li, Wei Gong, Meiyan Yang

**Affiliations:** 1 State Key Laboratory of Toxicology and Medical Countermeasures, Beijing Institute of Pharmacology and Toxicology, Beijing 100850, China; 2 School of Pharmacy, Guangxi Medical University, Nanning 530021, China

**Keywords:** antimicrobial resistance, multi-drug-resistant bacteria, antibiotics, antimicrobial materials, drug delivery systems

## Abstract

Bacterial infection is a common clinical disease. Antibiotics have saved countless lives since their discovery and are a powerful weapon in the fight against bacteria. However, with the widespread use of antibiotics, the problem of drug resistance now poses a great threat to human health. In recent years, studies have investigated approaches to combat bacterial resistance. Several antimicrobial materials and drug delivery systems have emerged as promising strategies. Nano-drug delivery systems for antibiotics can reduce the resistance to antibiotics and extend the lifespan of novel antibiotics, and they allow targeting drug delivery compared to conventional antibiotics. This review highlights the mechanistic insights of using different strategies to combat drug-resistant bacteria and summarizes the recent advancements in antimicrobial materials and drug delivery systems for different carriers. Furthermore, the fundamental properties of combating antimicrobial resistance are discussed, and the current challenges and future perspectives in this field are proposed.

## 1. Introduction

Bacterial infection is a common clinical disease that can affect a number of organs and tissues in the human body [1]. Antibiotics are used clinically to combat pathogenic bacteria, which in turn have gradually developed resistance to increasingly more antibiotics. Simultaneously, vancomycin, polymyxin and other antibiotics known as the “last line of defense” have also produced multi-drug-resistant (MDR) bacteria [2]. The accumulation of bacterial genetic mutations will lead to the emergence of “superbugs” and superbug infections that are almost incurable. This has made the treatment of clinical trauma infections extremely difficult, and scientists have speculated that mankind will soon enter the “post-antibiotic era” in response to the current situation [3].

Medical researchers have pointed out that about 50% of the world’s antibiotics are misused each year, and over 80,000 people in China currently die indirectly or directly from antibiotic misuse in China each year. The new Global Antimicrobial Resistance Surveillance System (GLASS) of the World Health Organization (WHO) has revealed widespread antibiotic resistance among 500,000 suspected bacterial infections in 22 countries [4,5]. In 2017, the WHO released the 12 most resistant “superbugs” that pose the greatest threat to human health, including carbapenem-resistant *Acinetobacter baumannii* (*A. baumannii*), *Pseudomonas aeruginosa* (*P. aeruginosa*) and *Escherichia coli* (*E. coli*), which are classified as “urgent” level and had the highest urgency for new antibiotics [6,7]. For example, *P. aeruginosa* displays an exceptional level of resistance to antibiotics and has the remarkable ability to develop antibiotic resistance in hospitalized patients [1].

**Figure 1:**
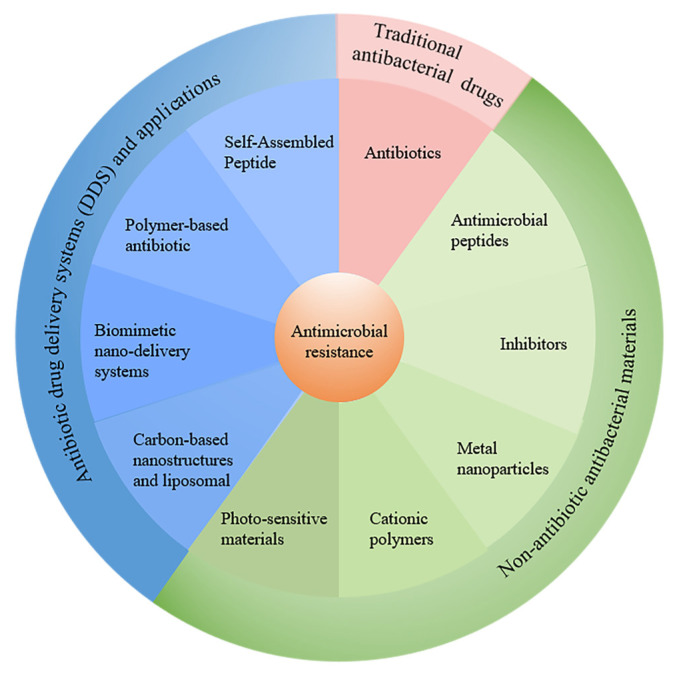
The recent strategies to combat antimicrobial resistance discussed in this review.

The number of deaths directly caused by antibiotic resistance in 2019 is equal to the number of deaths caused by AIDS and malaria combined, and antibiotic resistance-related deaths are the third leading cause of death globally after ischemic heart disease and stroke [5]. According to a recent survey by the Centers for Disease Control and Prevention (CDC), antibiotic resistance causes millions of infections around the world each year. The study estimated that by 2050, 10 million people worldwide each year will die due to bacterial resistance; this equates to one death every three seconds, which is higher than the current number of deaths from cancer [8]. Over the course of the global fight against the COVID-19 pandemic, there were increasing reports of bacterial infections that may have been common or secondary to respiratory infections in patients with COVID-19 [9]. In recent years, bacteria and other organisms have been detected in the microenvironment of various tumors, and studies have found that these bacteria are actually the “accomplices” of the tumors. It was found that most solid tumors, including breast cancer, lung cancer, melanoma and pancreatic cancer, contain bacteria, mostly tumor-specific intracellular bacteria [10]. Cai’s team at Westlake University reported that a variety of unique “intracellular bacteria” present in breast cancer tissues played an important role in the metastatic colonization process [11]. Bacteria have been constantly invading people, which means that we are facing a public health crisis of unimaginable proportions, and there is an urgent need for researchers to investigate new strategies and fight antimicrobial resistance (AMR) with new agents with lower drug resistance. In this review, we summarize the types of traditional antibiotics and their mechanisms of action and resistance. As conventional antibiotics are commonly used clinically and have been summarized in the relevant literature, we provide a brief overview of conventional antibiotics and instead focus on various other strategies to combat drug-resistant bacteria. In particular, strategies to combat the pressing bacterial resistance problem, including various antimicrobial materials and different drug delivery systems, are summarized and highlighted (Figure 1). Finally, we discuss the potential challenges of bacterial drug resistance and explore the development trends.

## 2. Traditional Antibacterial Drugs: A Brief Description

In 1928, British bacteriologist Alexander Fleming stumbled upon penicillin, the first antibiotic to be discovered by humans [12]. This discovery led to a revolution in the medical world, and humans were no longer helpless in the face of bacterial infections. Subsequently, antibiotics representing natural and chemically synthesized entities have become powerful tools in the fight against infectious diseases [13]. Antibiotics are commonly used in the treatment and prevention of infections and are classified according to their chemical structure (Table 1) [14].

Antibiotics have saved countless lives since their discovery, making them a powerful weapon in the fight against bacteria. However, antibiotics are not omnipotent. With the widespread use of antibiotics, the problem of drug resistance has gradually become serious [2]. Antibiotic resistance mechanisms are generated corresponding to their mechanism of action. The mechanisms of action and resistance of different types of antibiotics are summarized in the following sections.

### 2.1. Mechanisms of Antibiotic Action

Antibiotic-mediated cell death is a complex process that involves physical interactions between drug molecules and specific targets in bacteria and thus alters the state at the biochemical, molecular and ultrastructural levels in the affected bacteria. The mechanisms of action mainly include inhibition of the bacterial cell wall, protein and nucleic acid synthesis; changes to the cell membrane permeability; and inhibition of bacterial metabolic pathways [28].

**Figure 2:**
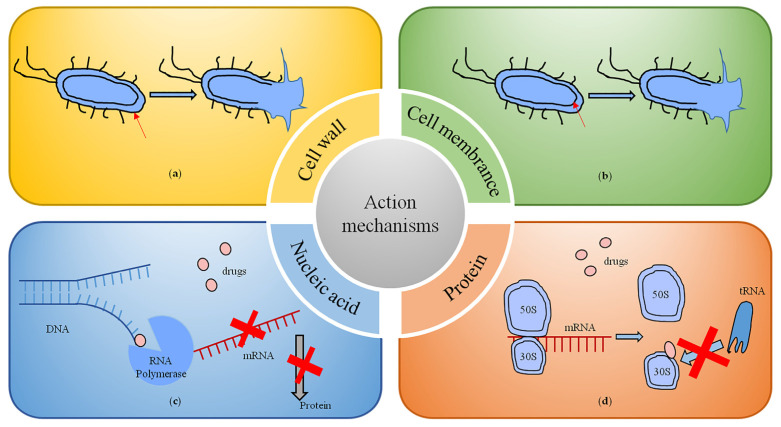
Mechanisms of antibiotic action. (**a**) Inhibition of bacterial cell wall synthesis. (**b**) The targets and interactions with bacterial cell membranes. (**c**) Inhibition of nucleic acid synthesis. (**d**) Inhibition of protein synthesis.

Inhibition of bacterial cell wall synthesis (Figure 2a) is the main action mechanism of β-lactam and glycopeptide antibiotics. The β-lactam antibiotics work by binding though the β-lactam ring to the bacterial penicillin-binding protein (PBP), which acts to synthesize and remodel bacterial peptidoglycans, thus inhibiting the transpeptidation effect [29]. The mechanism of action of vancomycin, a representative drug of glycopeptide antibiotics, is to form a hydrogen bond compound with the terminal dipeptide D-alanine-D-alanine region of the precursor lipid II of the peptidoglycan chain of the bacterial cell wall, interfering with the peptidoglycan layer maturation process and thereby preventing cell wall synthesis [30].

By altering the permeability of bacterial cell membranes, some antibiotics target and interact with cell membranes. Representative drugs are polymyxin antibiotics (Figure 2b). They act on the lipopolysaccharide on the outer membrane of Gram-negative bacteria and enter the periplasm after binding to the phosphate group of lipid A in the lipopolysaccharide, thus exerting their antibacterial effect. Polymyxins are commonly used in the treatment of infections caused by carbapenem-resistant bacteria such as *A. baumannii*, *E. coli* and *P. aeruginosa* [31].

Inhibition of nucleic acid synthesis, including RNA and DNA synthesis and DNA replication, is the main mechanism of action of rifamycin and quinolone antibiotics (Figure 2c). Quinolone antibiotics interfere with DNA superhelical changes by binding to topoisomerase II or topoisomerase IV, leading to double-stranded DNA breaks that induce cell death [32]. Rifamycin inhibits the activity of RNA polymerase in the bacterium by specifically binding to the DNA-dependent β-subunit of RNA polymerase, thereby impeding mRNA synthesis [33].

The drugs that inhibit bacterial growth by inhibiting protein synthesis are very extensive (Figure 2d), and include macrolides (e.g., erythromycin), lincosamides (e.g., clindamycin), aminoglycosides (e.g., streptomycin and gentamicin), amphetamines (e.g., chloramphenicol), oxazolidinones (e.g., linezolid) and tetracycline antibiotics [34]. The mechanism of action is mainly through the physical blockade of protein translation initiation or translocation of peptidyl tRNAs to interfere with the stability of peptidyl tRNA binding to ribosomes [29].

### 2.2. Mechanisms of Antibiotic Resistance

**Figure 3:**
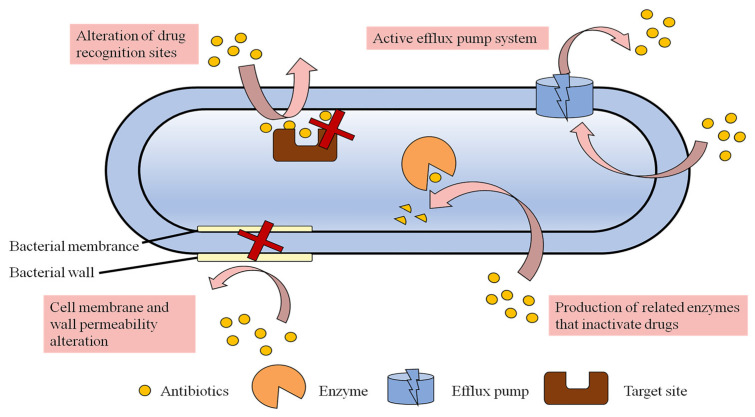
Mechanisms of antibiotic resistance.

In the process of anti-infection treatment, bacteria usually develop resistance to avoid being killed by antibiotics. Significantly, with the continued abuse and overuse of traditional antimicrobial drugs worldwide, bacterial tolerance and resistance are increasing at an alarming rate [35]. According to a report’s data, a new antibiotic can develop bacterial resistance within five years of entering the market for use [36]. In 2019, the WHO listed antimicrobial resistance as one of the top 10 threats to global health [37]. The failure of traditional antimicrobial drugs is a global public health problem that is further exacerbated by the increase in global antibiotic consumption [38]. At present, MDR bacteria have developed multiple resistance mechanisms to combat most of the available antibiotics. These resistance mechanisms can be explained from different perspectives, including genetic, metabolic and biochemical mechanisms. Genetic mechanisms refer to the fact that bacteria can develop resistance through various pathways, such as mutations of their own genes, vertical transmission of chromosomes, horizontal transmission of plasmids or transposons, and capture of exogenous resistance genes by integrons [13]. The metabolic mechanism at the physiological level mainly describes a “viable but non-culturable state” (VBNC) of bacteria, which is a unique survival strategy of bacteria under stress. In this state, bacterial growth and metabolism are very slow or even nearly cease, and most antibacterial drugs only kill metabolically active bacteria; therefore, these bacteria can escape the action of antibiotics and enter the latent phase [39,40]. The biochemical mechanisms at the protein level mainly reflect the tolerance of bacteria to antibiotics, in which bacteria produce and release functional proteins or alter their own structural proteins. A schematic of the four different mechanisms of drug resistance at the protein level is shown in Figure 3. The following section described these mechanisms in more detail.

#### 2.2.1. Cell Membrane Permeability Alteration

Bacteria have thick cell walls and cell membranes and can hinder the entry of drugs such as antibiotics by regulating the permeability of the cell membrane so that the uptake of drugs is decreased without reaching an effective lethal concentration. This non-specific resistance mechanism is mostly seen in Gram-negative bacteria due to the presence of outer membrane structures [36]. The outer lipopolysaccharide of Gram-negative bacteria is a tightly packed hydrophilic carbon and nitrogen molecule, which hinders the uptake of hydrophobic antibiotics and thus leads to drug absorption and resistance [41]. The main reasons for the decrease in outer membrane permeability include defects in membrane pore proteins, multidirectional mutations, specific channel alterations and lipid bilayer alterations, which prevent the entry of antibiotics [42]. For example, *P. aeruginosa* is resistant to imipenem due to the lack of D2 pore protein [43].

#### 2.2.2. Active Efflux Pump System

Efflux pumps are intact membrane proteins in the cell membrane that utilize metabolic energy to resist concentration gradients to expel drugs out of the cell, and are a class of energy-dependent transporter proteins [44]. This mechanism is prevalent in both Gram-negative and Gram-positive bacteria. These efflux pumps can be specific to particular compounds and can cause the amount of antibiotic remaining in the cell to be insufficient to reach an effective drug concentration, leading to strain resistance [36]. Transporter proteins that are capable of non-selectively expelling antibiotics from inside the cell are called MDR transporter proteins. They are important not only for antibiotic resistance but also for promoting bacterial membrane formation [45,46]. Multidrug exhaust systems (MESs) can be classified into the following six families based on their structure and energy requirements: (i) the major facilitator superfamily (MFS), (ii) the small multidrug resistance family (SMR), (iii) the multidrug and toxic compound extrusion family (MATE), (iv) the resistance–nodulation–cell division superfamily (RND), (v) the adenosine triphosphate (ATP)-binding cassette superfamily (ABC) and (vi) the proteobacterial antimicrobial compound efflux family (PACE) [47]. For example, the NorA efflux pump of *Staphylococcus aureus* (*S. aureus*) pumps quinolones out of the bacteria [48]. Multidrug resistance pumps can pump drugs out of the cell in a diverse manner, such as through AcrAB-TolC on the cell membrane of *E. coli* [40] and MexAB-OprM in *P. aeruginosa*. Furthermore, different types of antibiotics can be expelled by multidrug resistance pumps. For example, the RND efflux pump can pump macrolides, chloramphenicol, tetracyclines, quinolones and β-lactams out of the bacteria [36].

#### 2.2.3. Alteration of Drug Recognition Sites

A drug can bind specifically to the target site of proteins in bacteria and exert antibacterial effects. However, drug-resistant bacteria can reduce the binding of antibacterial drugs or render them ineffective by changing the target site or creating a new target site. For example, vancomycin resistance results from changes in the bacterial site of action of peptidoglycan precursors that reduce the binding of the drug to the bacteria, making vancomycin less effective [49]. Alterations in PBP can result in resistance to β-lactam antibiotics [50], and alterations in DNA topoisomerase can result in resistance to quinolone antibiotics [51].

#### 2.2.4. Production of Related Enzymes That Inactivate Drugs

Drug-resistant bacteria can produce enzymes that inactivate antibiotics, including modifying enzymes, hydrolytic enzymes, and passivating enzymes, as a plasmid- or chromosome-mediated mechanism [52]. The production of enzymes that decompose or inactivate antibiotics, leading to the destruction or inactivation of one or more antimicrobial drugs, is an important cause of bacterial resistance. A classic example of this resistance mechanism is β-lactamases, which can disrupt the amide bond in the ring structure of β-lactam drugs and render the antibiotic ineffective. This is considered to be the most common resistance mechanism leading to β-lactam resistance in Gram-negative bacteria [53]. In addition, there are many enzyme-mediated modification mechanisms of bacterial resistance in aminoglycoside antibiotics. The modification enzymes, such as acetyl and nucleoside phosphotransferases, can modify aminoglycoside antibiotics by N-acetylation, O-phosphorylation and O-adenylation, leading to the loss of antibacterial activity due to the disappearance of ribosomal targets [54]. For example, MDR bacteria can produce multiple different types of enzymes that are broadly resistant to multiple antibiotics, such as methicillin-resistant *Staphylococcus aureus* (MRSA) [55].

## 3. Antimicrobial Materials

According to statistics, most of the available antibiotics approved in recent years were based on discoveries made prior to 2010. The undeniable importance of AMR has led to a global call for the urgent discovery of new antibiotics and treatments. However, the problem of antibiotic resistance cannot be solved once and for all, and the development of new antimicrobial agents or antimicrobial strategies in the fight against microorganisms is destined to be a perpetual process [56]. In recent years, a research boom in non-traditional antibiotic treatment strategies combating bacterial resistance has emerged, and this article reviews the selection and application of strategies to meet these challenges.

### 3.1. Antimicrobial Peptides (AMPs)

Usually, traditional single-target antibiotics are prone to resistance after long-term extensive use [57]. As a novel alternative to traditional antibiotics, AMPs have shown powerful broad-spectrum antimicrobial characteristics against a range of MDR pathogens [58], and resistance has minimally developed or has not occurred at all, due to the specific bactericidal multi-modal action mechanisms of AMPs.

In 1980, Swiss professor G. Boman isolated the world’s first animal AMP cecropins from a chrysalis. Since then, a variety of antimicrobial peptides with antimicrobial activity have been discovered, designed and modified. AMP research has become a hot spot in the fight against bacterial resistance in recent years. For example, Breij et al. designed and synthesized a series of antimicrobial and antibiofilm peptide SAAPs using human antimicrobial peptide LL-37 as the parent peptide, and the antimicrobial activity was effectively enhanced in comparison with LL-37. Among these, SAAP-148 was found to be a successful agent in destroying MDR pathogens, blocking biofilm development and eliminating pre-existing biofilms and persister cells [59]. Furthermore, Yue Fei et al. designed an AMP (FOTyr-AMP) based on the potential ability of nitric oxide to disperse biofilms and its unique antimicrobial activity. The chemical combination of nitric oxide and antimicrobial peptides enabled the peptide to achieve the dual biological functions of bacterial biofilm removal and potent antimicrobial activity, and the effect was superior to that of cephalosporin C [60].

Among the rapidly developing AMPs, proline-rich AMP (PrAMP) with low toxicity and multiple intracellular targets is considered as a particularly promising candidate for rational design to target Gram-negative pathogens. Li et al. obtained the proline-rich AMP monomer Chex1-Arg20 through different chemical modifications to broaden its antimicrobial spectrum. The team then used two bifunctional connectors, tetrafluorobenzene and octafluorobiphenyl, to dimerize Chex1-Arg20, which significantly enhanced the antimicrobial activity of the monomer Chex1-Arg20 [61]. The resulting dimeric peptide showed excellent potency against Gram-negative bacteria, especially the WHO-listed MDR *A. baumannii*, with no cytotoxicity. Moreover, a time-kill kinetic assay revealed that the dimeric peptide killed bacteria rapidly and was able to reduce the preformed bacterial biofilm by more than 50%, which provided key parameters for further clinical pharmacokinetics and drug development, with potential for therapeutic application [62].

**Figure 4:**
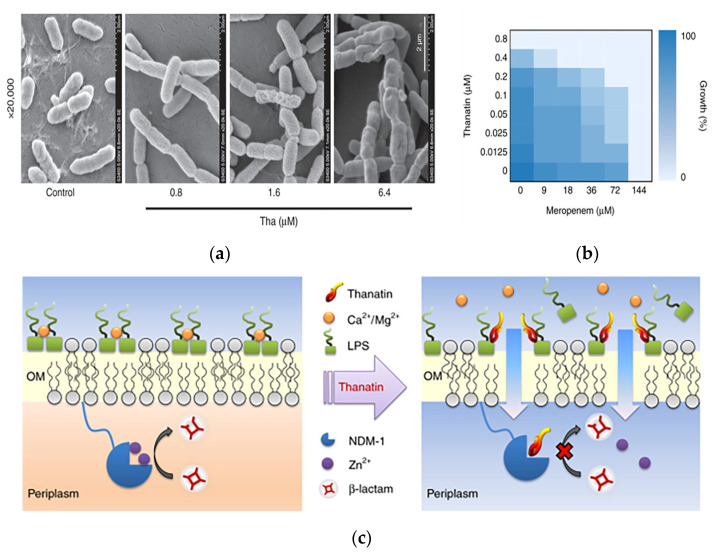
A novel chemically synthesized AMP against drug-resistant bacteria in antimicrobial materials. (**a**) Morphology of *E. coli* XJ141026 was investigated by scanning electron microscopy 4 h after thanatin treatment. Scale bar: 2 μm. (**b**) Thanatin rescues the activity of carbapenem. Microdilution checkerboard analysis showing the combined effect of thanatin and meropenem against NDM-1-producing *E. coli* XJ141026. The heat plot shows an average of three technical replicates. (**c**) Mechanism of action of thanatin. Adapted with permission from [64], Copyright 2019 Springer Nature.

In addition to acting as bactericidal molecules alone, AMPs can also act as antibiotic adjuvants, enhancing the efficacy of antibiotics through a synergistic strategy. Song et al. identified a short linear AMP named (SLAP)-S25, which has only weak antimicrobial activity on its own but triggered membrane damage by binding to lipopolysaccharides on the outer membrane of Gram-negative bacteria and phosphatidylglycerol (PG) in the bacterial plasma membrane, thereby assisting the efficacy of a variety of other antibiotics, including cefepime, colistin, ofloxacin, rifampicin, tetracycline and vancomycin, in the intracellular compartment where they accumulated and exerted bactericidal efficacy [63]. Ma et al. showed that the AMP thanatin induced the release of lipopolysaccharide by competitively displacing divalent cations from the bacterial outer membrane, thereby disrupting the outer membrane of New Delhi metallo-β-lactamase-1 (NDM-1)-producing bacteria. The action of thanatin on the outer membrane of the bacteria was observed in scanning electron microscopy images, in which the cells exhibited increased corrugation on the surface as the concentration of thanatin increased (Figure 4a). Furthermore, thanatin inhibited the enzymatic activity of NDM-1 by displacing zinc ions from the active site, thereby reversing carbapenem resistance in NDM-1-producing bacteria in vitro and in vivo. The capacity of NDM-1 inhibition suggested that thanatin could potentially protect conventional antibiotics from hydrolysis and restore the antibiotic susceptibility of NDM-1-producing strains. The peptide was shown to restore the antibacterial activity of meropenem against drug-resistant *E. coli*, with the minimum inhibitory concentration (MIC) value decreasing from 144 μM to 18 μM (Figure 4b). The dual mechanism of action of this antimicrobial peptide not only allows the disruption of the bacterial outer membrane but also successfully reverses bacterial resistance, which is a major advantage compared to antibiotics (Figure 4c). As such, thanatin is a promising candidate to combat the emergence and dissemination of NDM-1-producing bacteria [64].

AMPs have broad application prospects due to their high antimicrobial activity, broad antimicrobial spectrum and wide variety, and because target strains are less likely to produce resistance mutations. At present, the peptide antimicrobial drugs that have been marketed for therapeutic use include short bacillus peptide and polymyxin [31]. In addition, many patents have been reported on the effective sequences of antimicrobial peptides. Although there are many studies on AMPs, very few AMPs are used in clinical treatment. Most of them are still in phase II and III clinical trials, and there are still many problems to be solved in the application and production of AMPs. For example, the chemical structure of antimicrobial peptides is a peptide-like molecule, and therefore they are easily degraded by enzymes. With regard to natural antimicrobial peptides, the resources are limited and the extraction process is complicated. For chemically synthesized peptides, the high cost and difficulties of industrialization should be considered.

### 3.2. Inhibitors as Adjuvants

Inhibitors are often used as adjuvants to enhance the effectiveness of antibiotics. For example, β-lactamase inhibitors usually act in combination with β-lactam antibiotics by binding to the active site of β-lactamase, effectively inhibiting the β-lactamase, which prevents the lactam ring in the antibiotic from being hydrolyzed, thus maintaining the structural integrity of the antibiotic and producing an antibacterial effect. In the face of bacterial resistance, inhibitors targeting enzymes and efflux pumps produced by drug-resistant bacteria for the study of anti-drug-resistant bacterial drug candidates are still very effective at this stage.

#### 3.2.1. Enzyme Inhibitors

**Figure 5:**
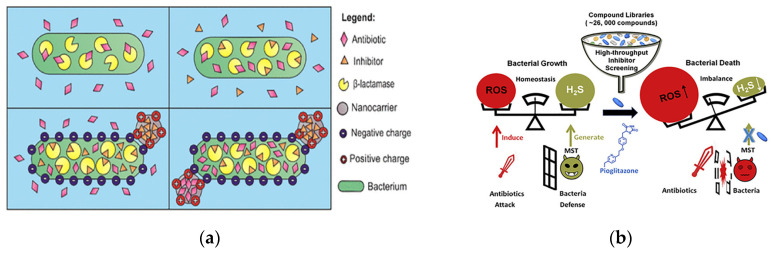
Antibacterial modalities of enzyme inhibitors. (**a**) Schematic of clavulanic acid loaded in functionalized nanocarriers. Adapted with permission from [66], Copyright 2022 American Chemical Society. (**b**) Enzyme inhibitors improve the killing effect on bacteria by disrupting the ROS balance for cell survival. Adapted with permission from [68], Copyright 2020 Elsevier.

For resistant bacteria that produce enzymes and thus render antibiotics ineffective, finding and developing appropriate enzyme inhibitors can solve the primary cause of the drug resistance problem. Clavulanic acid, the first broad-spectrum β-lactamase inhibitor in clinical use, has minimal antibiotic efficacy despite its β-lactam ring structure. In combination with other antibiotics, clavulanic acid enhanced the efficacy of the antibiotics [65]. Clavulanic acid penetrated the cell walls of several bacteria more effectively (2 to 25 times) than other β-lactamase inhibitors. This led to good therapeutic efficacy with a combination of β-lactam antibiotics and clavulanic acid against β-lactam-resistant bacteria (Figure 5a) [66]. Amoxicillin–clavulanic acid was the first combination of β-lactam and β-lactamase inhibitors to be used clinically since 1981 and remains the only available oral formulation in this category [67].

NDM-1 is one of the β-lactamases, and Sun’s team found that Au in the antirheumatic drug auranofin replaced zinc in the active site of NDM-1, thus inactivating NDM-1. The results showed that auranofin substantially reduced the MIC of meropenem in NDM-1-producing *E. coli*, with a fractional inhibitory concentration index (FICI) of 0.156. For mobilized colistin resistance-1 (MCR-1)-producing bacteria, the combination of auranofin and polymyxin substantially reduced the MIC of polymyxin, with a FICI of about 0.125. This also provides a potential therapeutic strategy for clinical treatment by combining auranofin with carbapenem antibiotics to restore the antibiotic action against superbugs that carry multiple resistance genes [69]. Omar M. El-Halfawy et al. identified a potent bioactive substance, MAC-545496, based on a cascade of high-throughput screening platforms, that reversed the resistance of MRSA to β-lactam drugs. It was shown that the β-lactam resistance of MRSA strains was reversed using 0.06 μg/mL of MAC-545496, and the MIC of cefuroxime against *S. aureus* was reduced from 512 μg/mL to 8 μg/mL using 0.03 μg/mL of MAC-545496. In addition, MAC-545496, together with cefuroxime and oxacillin, was shown to work against more than 10 strains in clinical isolates of *S. aureus* [70].

Reactive small molecules of hydrogen sulfide (H_2_S) have the ability to protect bacteria from oxidative stress, and H_2_S-producing enzymes can antagonize the antibacterial effect of antibiotics by producing H_2_S. By constructing a high-throughput drug screening model for *E. coli* 3-mercaptopyruvate transsulfurase (eMST), Giorgia Croppi et al. identified the first active inhibitor of eMST, pioglitazone (an FDA-approved drug for the treatment of type II diabetes), from more than 26,000 compounds. They systematically investigated the mechanism of action of this inhibitor at the molecular and bacterial level and revealed that it enhanced the bactericidal effect of antibiotics (Figure 5b) [68]. Shatalin et al. performed a protein structure-based screening of bacterial hydrogen sulfide-producing enzymes for inhibitors and identified a set of bacterial cystathionine γ–lyase (bCSE) inhibitors that acted through a metamorphic mechanism. They demonstrated the key role of bCSE in H_2_S biogenesis in *S. aureus* and *P. aeruginosa* and synthesized bCSE inhibitors that enhanced a variety of bactericidal antibiotics. Their experimental data suggested that resistant bacteria produced more H_2_S than non-resistant bacteria. They found that by using bCSE inhibitors to interfere with the ability of bacteria to resist drugs, combined antibiotic use had the potential to reduce treatment failure rates in acute infections [71].

#### 3.2.2. Efflux Pump Inhibitors (EPIs)

Efflux pumps are important for both intrinsic and acquired resistance of bacteria. Identifying and inhibiting EPIs to restore the effectiveness of existing antibiotics is an active area of research. Over the past 20 years, significant efforts have been made to identify novel EPIs.

**Figure 6:**
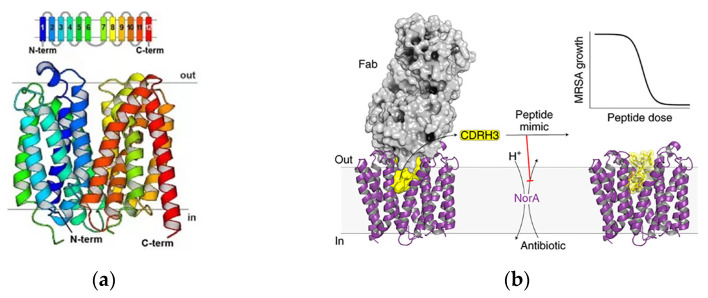

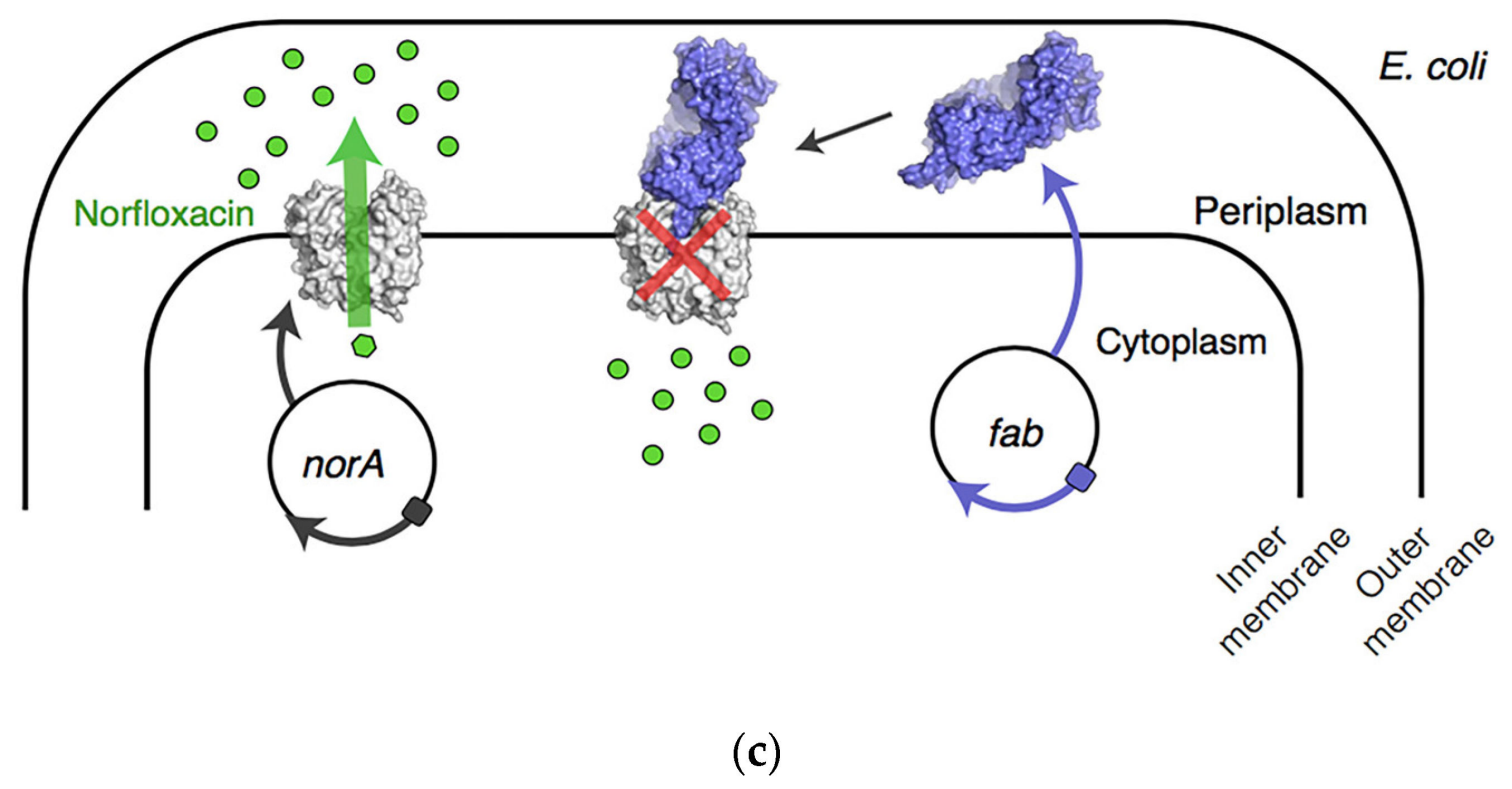
Inhibitors of the NorA efflux pump. (**a**) NorA topology and structure. The TM helices of NorA are colored in rainbow. TM1–TM6 and TM7–TM12 define the N and C domains of the transporter, respectively. (**b**) Schematic of the structure of the inhibitor bound to the substrate pocket. (**c**) Schematic of the mechanism of action of the Fab ring reversal for the restoration of antibiotic activity in drug-resistant bacteria. Adapted with permission from [72], Copyright 2022 Springer Nature.

NorA is a chromosomally encoded multidrug efflux pump in MRSA, and the structure is shown in Figure 6a. NorA is comprised of 12-transmembrane (TM) α-helices arranged in two 6-TM bundles (N- and C-terminal domains) that straddle a putative substrate-binding pocket. Douglas N et al. identified synthetic antigen-binding fragment Fabs by structural analysis of the substrate binding pocket that bound to NorA. The Fab ring was inserted into the substrate binding pocket of NorA to inhibit antimicrobial drug pumping and thus addressed the bacterial resistance problems. The portion inserted into the substrate binding pocket was CDRH3 (Figure 6b). The study showed that submicromolar concentration of the Fab ring of peptide mimics inhibited NorA and inhibited the growth of MRSA in combination with the antibiotic norfloxacin (Figure 6c). The Fab CDRH3 loop was observed to be inserted into the substrate binding pocket of NorA, indicating that Fab binding inhibited antibiotic efflux [72].

Felicetti synthesized and screened a series of methoxy-2-phenylquinoline derivatives. Among the 35 different derivatives synthesized, compounds 3b and 7d showed good NorA inhibitory activity by reducing the MIC of ciprofloxacin against drug-resistant *S. aureus* at very low concentrations. Importantly, these two compounds showed EPI activity at concentrations that were non-toxic to human cells and showed promise for good therapeutic efficacy, according to preliminary pharmacokinetic studies [73]. In additional studies on Gram-negative bacteria efflux pump inhibitors, Grimsey et al. provided experimental evidence that the antipsychotic drugs chlorpromazine and amitriptyline are inhibitors of the AcrB transporter, the main RND efflux pump in *E. coli* and *Salmonella typhimurium* [74].

### 3.3. Metal Nanomaterials

Studies have shown that metal nanomaterials such as gold, silver and zinc can be used directly for the detection and treatment of bacterial infections, in addition to AMPs. The antimicrobial properties of metal nanomaterials depend largely on the size, shape and composition of the formed nanoparticles. Metal nanoparticles have shown promising prospects in nanomedicine research due to their physicochemical properties. Among metal nanomaterials, silver nanoparticles (Ag-NPs) have gained great interest in drug delivery, imaging and biosensing, and antimicrobial wound dressings [75]. ZnO-NPs, a ZnO nanomaterial, showed antibacterial activity against both Gram-negative (*E. coli*) and Gram-positive (*S. aureus*) bacteria in antibacterial assay testing [76]. It was found that bacteria treated with Au-NPs at a concentration of 33% of the MIC remained susceptible to Au-NPs under MIC treatment after 30 days of continuous passages, demonstrating that Au-NPs are not susceptible to bacterial drug resistance [77].

### 3.4. Cationic Polymers

Cationic polymers with intrinsic antibacterial activity include polyquaternary ammonium salts (PQASs), chitosan, polyethyleneimine (PEI) and their derivatives. Their positive charge interacts with the negative charge of the bacterial surface, causing destruction of the bacterial cell membrane or cell wall structure, extravasation of cytoplasm and ultimately bacterial death.

PQASs can be prepared by polymerization of small molecule quaternary ammonium compounds or quaternization of polymers. For example, Lv et al. prepared poly([2-(methacryloyloxy)-ethyl] trimethyl ammonium chloride (METAC) nanofiber membranes by in situ cross-linking polymerization using [2-(methacryloyloxy)-ethyl] trimethyl ammonium chloride(PMETAC), 2,2′-azobisisisobutyronitrile (AIBN) as the initiator and N,N′-methylenebisacrylamide (MBA) as the cross-linker. All the PMETAC-decorated nanofibrous membranes (NFMs) showed high antibacterial ratios as high as 90% for both *E. coli* and *S. aureus*. Impressively, the NFM-6 showed a nearly 99% bacterial killing ratio for both *E. coli* and *S. aureus* [78].

Chitosan is a natural polymer material with good biocompatibility, biodegradability and antibacterial properties. Min et al. combined quaternary ammonium salt-modified chitosan (HACC) with polyvinyl alcohol (PVA) to obtain a composite coating material. The HACC gave the coating surface an excellent antimicrobial ability with a killing rate for bacteria, such as *E. coli* and *S. aureus*, of up to 99% [79].

PEIs have a wide range of applications in the antimicrobial field due to their high density of positive charges and diverse structures [80]. It has been shown that cationic PEI nanoparticles as antimicrobial agents can effectively bind to proteins and stabilize their structures [81]. Beyth et al. incorporated cross-linked quaternary ammonium polyethylene-diamine (QPEI) nanoparticles into dental resin composites, giving them a long-lasting, broad antimicrobial effect with no measurable side effects in in vitro biocompatibility experiments [82].

### 3.5. Photo-Sensitive Materials

Photo-sensitive materials combined with photodynamic therapy can induce cellular and microbial inactivation, which is called photodynamic antimicrobial chemotherapy (PACT). PACT is an oxidative damage mechanism based on the synergistic action of three factors: light, a photosensitizer and oxygen. The mechanism rarely leads to bacterial resistance of primary bacteria or offspring after several differential passages of the remaining bacteria [83]. PACT has received widespread attention in the antimicrobial field because of its high spatiotemporal selectivity, noninvasiveness, low incidence of drug resistance, and suitability for localized infection treatment due to the penetrating laser. Recently, a novel antimicrobial strategy, namely electroluminescent power therapy (ELDT), based on nano-assemblies of electroluminescent (EL) materials and photosensitizers, generated reactive oxygen species (ROS) in situ under an electric field, i.e., the fluorescence emitted by EL molecules excited the photosensitizers to produce singlet oxygen (^1^O_2_), which in turn caused oxidative damage. Zhang et al. prepared a flexible therapeutic device by integrating hydrogel-loaded ELDT nanoparticles. It was shown that the ELDT-based flexible device was able to exhibit a 99.9% antibacterial effect against drug-resistant bacteria through ROS-induced killing for superficial infection treatment and wound healing promotion [84].

## 4. Drug Delivery Systems

Systemic administration of antibiotics commonly has disadvantages, such as low bioavailability, side effects and antibiotic resistance. To enhance antibiotic biodistribution and bioavailability, drug delivery systems (DDSs) for antibiotics are a practical strategy to reduce the resistance of antibiotics and extend the lifespan of novel antibiotics. Scientists have proposed a “Trojan horse” strategy in the design and development of DDSs. The core of this strategy is to combine antimicrobial agents with different types of carriers (e.g., liposomes, erythrocytes, exosomes, polymers and self-assembled peptides) to achieve efficient drug delivery. The efficient delivery of drugs is achieved by breaking the resistance barrier created by drug-resistant bacteria [85]. In addition, drug release at the infection site can be achieved using DDSs by targeting the unique microenvironment associated with the infected tissue or by guidance from external stimuli.

### 4.1. Carbon-Based Nano-Delivery Systems

According to recent reports, carbon-based nanomaterials such as fullerenes, carbon nanotubes (CNTs) and graphene oxide (GO) nanoparticles have shown powerful antibacterial properties [86]. Meanwhile, due to their special structural characteristics and physicochemical properties, carbon-based nanomaterials not only have high antimicrobial activity, but also have good biocompatibility and environmental friendliness. Carbon-based nanostructures can come into direct contact with bacteria, thereby disrupting their cell membrane integrity, metabolic processes and morphology. 

Functionalized carbon-based nanomaterials as carriers of common antibiotics can reduce antibiotic resistance, improve the bioavailability of antibiotics and provide targeted delivery of antibiotics [87]. Sahar E. et al. investigated the antibacterial activity of functionalized multi-walled carbon nanotubes (F-MWNTs). The results showed significant antimicrobial activity against both *E. coli* and *S. aureus*. F-MWNTs were able to biologically isolate bacteria from their microenvironment, promote the development of toxic substances and expose cells to oxidative stress leading to bacteria death. The efficiency of F-MWNTs showed an enhanced inhibitory effect compared to commonly used antibiotics, with percentages reaching 85% [88]. Jiang et al. constructed polyethyleneimine (PEI)-modified graphene oxide (GO) as a carrier and release platform (pGO-TCH) for tetracycline hydrochloride (TCH). Structural characterization showed that TCH was uniformly and compactly deposited on PEI-modified GO nanosheets. pGO-TCH was evaluated for its antibacterial activity against *S. aureus* and *E. coli* and compared with pGO and TCH alone. pGO showed no antibacterial activity, while the MIC values of pGO-TCH nanoflakes for both strains were 4-fold lower than those of TCH alone. The nanoflakes could trap and destroy bacteria, and upon release, TCH could act on the bacteriophage ribosomal targets [89].

However, there are still many problems to overcome in the application and dissemination of carbon-based nano-drug delivery systems, such as high production costs, the system’s own defects and safety. However, compared to other inorganic metallic nanomaterials, carbon-based nanomaterials have a higher biosafety profile, but their toxicity is still one of the inevitable drawbacks [90]. Furthermore, further research is necessary to understand the exact mechanism of the antimicrobial activity of carbon nanostructures, and the issue of degradation as inorganic materials in vivo remains a key consideration.

### 4.2. Liposomal Drug Delivery Systems

Liposomes are one of the most widely studied nano-DDSs with good biocompatibility and modifiability, and antibiotic liposomes have entered clinical studies and have been marketed [91]. Liposomes are spherical vesicles formed by self-assembly of amphiphilic lipid molecules with a bilayer structure, with particle sizes ranging from 20 nm to 10 μm. Hydrophilic and amphiphilic molecules can be encapsulated in the core, whereas hydrophobic molecules can be partitioned into lipid bilayers for selective loading of drugs [92]. The unique properties of liposomes, such as non-immunogenicity, low toxicity and biofilm matrix–cell membrane fusogenicity, significantly improve the effectiveness of antimicrobial agents and reduce the recurrence of infections [93]. For example, Du et al. devised multifunctional double-crowned vesicles loaded with antibiotics. The positively charged vesicles treated periodontitis by crossing the biofilm to effectively deliver antibiotics. In addition to the deeper penetration of the vesicles into the biofilm, the antibacterial polypeptide chains on the vesicles and the drug ciprofloxacin worked together to kill bacteria and thus were found to be more effective than a single mechanism [94]. Gao proposed integrating RvD1 and ceftazidime antibiotics into human neutrophil membrane-derived nanovesicles that specifically targeted inflammatory vessels for the treatment of pulmonary infections caused by *P. aeruginosa* [95]. Using liposomes as antibiotic drug carriers can effectively increase the local concentration of antibiotics at inflammation sites, inhibit the development of bacterial-induced resistance, and facilitate the reduction of the systemic administration dose, reducing toxic side effects [96] (Table 2).

### 4.3. Biomimetic Nano-Delivery Systems

Biomimetic nano-delivery systems are a relatively novel biological delivery strategy with properties such as long circulation, focal site targeting and immune escape. In recent years, cell membrane-encapsulated carriers have been increasingly used in the biomedical field.

#### 4.3.1. Erythrocytes

Erythrocytes, which are red blood cells (RBCs), can camouflage nanoparticles. Erythrocyte membrane nanovesicles can be used as a carrier system for delivering drugs, enzymes, peptides and antigens in vivo. Erythrocytes as human components have the advantages of good biocompatibility, a long circulation cycle, and high targeting, due to the intact retention of the structure and surface proteins of the erythrocyte membrane. Accordingly, erythrocyte membrane-encapsulated nanoparticles have become a potential nano-drug delivery platform in recent years.

**Figure 7:**
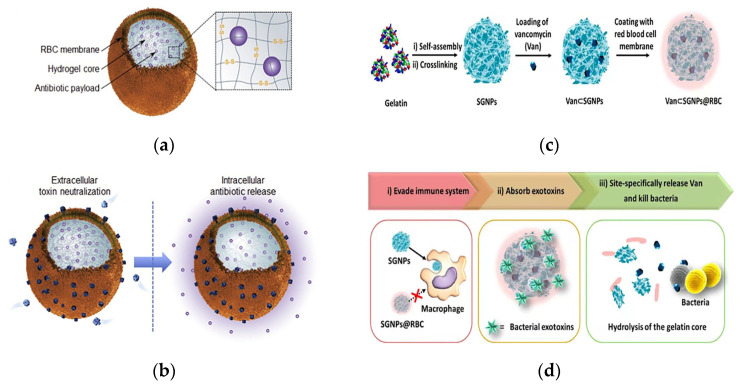
Schematic of an erythrocyte membrane-encapsulated drug delivery system. (**a**) Schematic of the structural composition of the RBC-nanogel DDS. (**b**) Mechanism of action of the RBC-nanogel DDS. Adapted with permission from [105], Copyright 2017 Elsevier. (**c**) The preparation process of the Van⊂SGNPs@RBC. (**d**) Schematic representation of adaptive and multifunctional Van⊂SGNPs@RBC in the treatment of a bacterial infection. Adapted with permission from [106], Copyright 2014 American Chemical Society.

Notably, a key factor in MRSA infection is the virulence caused by the various pore-forming toxins (PFTs) secreted by the bacteria [104]. Zhang’s team used strategies that targeted bacteria and neutralized bacterial toxin factors to address the problem of antibiotic resistance. They reported an erythrocyte membrane-coated nanogel (RBC-nanogel) system with combined antiviral and reactive antibiotic delivery for the treatment of MRSA infection. The delivery system consisted of a responsive hydrogel core, an RBC membrane shell and an antibiotic payload (Figure 7a). The RBC membrane was encapsulated on the nanogel through an in situ gelation process of membrane vesicle templating, whereas redox reactivity was achieved through a disulfide bond-based cross-linking agent. The RBC-nanogels were shown to effectively neutralize MRSA-associated toxins in the extracellular environment, and the neutralization of toxins in turn promoted bacterial uptake by macrophages. Once inside the cell, the RBC-nanogels showed accelerated drug release, which resulted in more effective bacterial inhibition (Figure 7b). When added to macrophages infected with intracellular MRSA bacteria, RBC-nanogels significantly inhibited bacterial growth in comparison with their free antibiotic and unresponsive nanogel counterparts. These results suggested that the RBC-nanogel system has great potential as a new and effective antibacterial agent against MRSA infection [105].

Li et al. proposed a core–shell supramolecular gelatin nanoparticle (SGNP)-based antibiotic delivery system, Van⊂SGNPs@RBC, for delivery of antibiotics on-demand. Bacterial infection sites self-assemble into SGNPs in the presence of gelatinase. The surface of the SGNPs was modified with erythrocyte membranes (SGNPs@RBCs), and vancomycin was further encapsulated in SGNPs@RBCs (Figure 7c). The coating of the RBC membrane imparted bionic properties and significantly enhanced the immune evasion capacity of the nanocarriers, which effectively accumulated at the site of infection through enhanced permeability and retention effects. Upon reaching the infected microenvironment, Van⊂SGNPs@RBCs were able to further absorb the exotoxins and alleviate the symptoms caused by the bacteria. At the same time, the gelatin nuclei were degraded by gelatinases in the infected microenvironment, and the encapsulated vancomycin was subsequently released and locally killed the pathogenic bacteria (Figure 7d). This approach demonstrated an innovative, bionic antibiotic delivery system that treated bacterial infections with minimal antibiotic dosing [106].

#### 4.3.2. Exosomes

In the last few years, exosomes have attracted considerable attention as drug delivery vehicles. Exosomes are intracellular membrane-based vesicles secreted by almost all types of cells. Exosomes play a crucial role in intercellular communication and are effective in delivering drugs to receptor cells [107,108]. Yang et al. reported a novel exosome-based antibiotic delivery system for the eradication of intracellular MRSA in which mannosylated exosomes (MEXos) were used as drug carriers that were preferentially engulfed by macrophages and subsequently delivered lysozyme (MEXoL) and vancomycin (MEXoV) to intracellular pathogens. In addition, MExos rapidly accumulated in the liver and spleen, which are the target organs of intracellular MRSA in mice, after intravenous administration. Thus, the MExos antibiotic delivery system is a promising strategy to combat intracellular infections [109].

### 4.4. Polymer-Based Antibiotic Delivery Systems (PADSs)

PADSs can protect antibiotics from premature metabolism and optimize their pharmacokinetics, and can achieve active targeting through specific ligand–receptor interactions or passive targeting through internal and external environmental responses. To date, several PADSs have been reported, such as polymeric liposomes, polymeric micelles, highly branched polymers and dendrimers, and polymeric nanogels. PADSs have shown enhanced therapeutic effects compared to free antibiotics [110]. Antimicrobial polymers are of great interest because of their low cost, simple preparation, antimicrobial effect and easy modifications [111].

**Figure 8:**
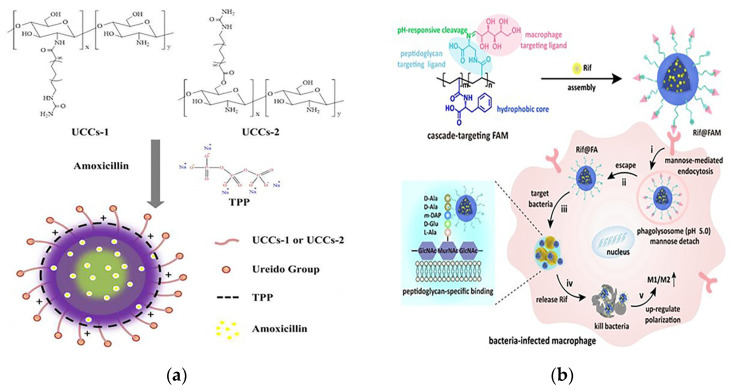
(**a**) Conformation diagram of the prepared amoxicillin-loaded ureido-conjugated chitosan/TPP nanoparticles. Adapted with permission from [112], Copyright 2016 Elsevier. (**b**) Schematic of the molecular structure and mechanism of action of the cascade targeted delivery system. Adapted with permission from [113], Copyright 2022 John Wiley and Sons.

*Helicobacter pylori* (*H. pylori*) is the culprit of gastric ulcers. Amoxicillin is a traditional drug against *H. pylori*, but it has low efficacy due to the short retention time in gastric mucosa. Jing et al. prepared multifunctional nanoparticles loaded with amoxicillin using covalently modified ureido-conjugated chitosan/tripolyphosphate (UCC/TPP), as shown in Figure 8a. Chitosan has excellent biocompatibility, biodegradability and antibacterial activity, making it promising as a biomaterial for delivering drugs. With the help of the deprotonating effect of pH-sensitive chitosan at higher pH microenvironments, the controlled release of the drug only at the *H. pylori* infection site through the dissolution of the nanoparticles was achieved, and thus the effectiveness of the antibiotics was increased. The results showed that amoxicillin UCC/TPP nanoparticles had excellent pH-sensitive properties, which delayed the release of amoxicillin in gastric acid and enabled effective targeted delivery of the drug in the survival zone of *H. pylori.* Compared with unmodified UCC/TPP nanoparticles, amoxicillin UCC/TPP nanoparticles showed a more specific and effective inhibitory effect on the growth of *H. pylori* [112].

Paunov et al. demonstrated a new nanotechnology-based cationic nanocarrier antimicrobial agent loaded with a β-lactamase inhibitor (clavulanic acid) and a β-lactam antibiotic (amoxicillin or ticarcillin) to overcome β-lactamase antimicrobial resistance. They created surface-functionalized shellac/poloxamer 407 stable antibiotic nanocarriers against *P. aeruginosa*. The amplification of the antibiotic effect of amoxicillin and ticarcillin loaded in the shellac nanoparticles, either alone or co-treated with free or nanocarrier-loaded clavulanic acid, was demonstrated. This study also reported a significant increase in the antimicrobial effects of clavulanic acid loading in these nanocarriers as a cotreatment. This was due to the increased antimicrobial activity of cation-functionalized antibiotic-loaded nanoparticles with electrostatic attraction to the bacterial cell wall, thus providing higher local concentrations of antibiotics and inhibitors. This effect was attributed to the accumulation of clavulanic acid-loaded nanocarriers on the bacterial cell wall, which allowed a higher percentage of inhibitors to interfere with the intracellular β-lactamase, thereby enhancing the antimicrobial efficacy [66].

Intracellular bacteria in latent or dormant states are resistant to high doses of antibiotics, and combating these opportunistic bacteria has been a long-standing challenge. To address the problem of high failure rates of the clinical clearance of intracellular bacteria, Feng’s team designed a cascade-targeted drug delivery system that sequentially targeted macrophages and intracellular bacteria and then achieved in situ drug delivery around the bacteria for precise release of antibiotics to overcome antibiotic tolerance. The DDS was fabricated by encapsulating rifampicin (Rif) into mannose-decorated poly(α-N-acryloyl-phenylalanine) (PF)-block-poly(β-N-acryloyl-D-aminoalanine) (PA) nanoparticles, denoted as Rif@FAM NPs. The PF in the structure formed a hydrophobic nucleus, PA was the bacterial targeting ligand and mannose acted as a macrophage targeting ligand. Rif@FAM NPs as a cascade targeting system achieved the effect of killing intracellular bacteria by the following steps: First, selective access to macrophages was achieved through mannose-mediated endocytosis. Subsequently, mannose was isolated under acidic conditions provided by phagosomes. The exposed D-aminoalanine fraction contributed to the escape of Rif@FA NPs into the cytoplasm. At this point, the Rif@FA NPs specifically bound to peptidoglycans on the membrane of Gram-positive bacteria, anchoring them to the intracellular bacteria, and then Rif was released to kill the intracellular bacteria. Elimination of bacteria was accompanied by upregulation of immune M1/M2 polarization in vivo (Figure 8b). Its elimination efficiency in vitro and in vivo was superior to that of free Rif or DDSs without targeting moieties, providing a new strategy for the treatment of intracellular bacterial infections [113].

### 4.5. Self-Assembled Peptide Drug Delivery Systems

Despite the strong advantages of each of the delivery systems reviewed above, the complex in vivo environment can cause many problems, such as the penetration ability of larger or more complex delivery systems and their degradation by the in vivo microenvironment. Therefore, self-assembly is a powerful tool for the preparation and delivery of polymeric materials [114]. Currently, peptide-based self-assembled nanostructures with higher stability and biocompatibility than free peptides have promising applications in the antibacterial DDS field [115]. It has been found that self-assembled peptides can form defined and regular aggregates with extensive functions under specific conditions. Recently, researchers have proposed a new strategy, in vivo self-assembly, which is the in situ construction of peptide-based nanomaterials in vivo, to counteract the ex vivo construction challenges of nanomaterials for biomedical applications [116]. Nanostructured peptides formed by peptide self-assembly generally rely on their own amphiphilic and positive electrical properties close to the bacterial cell membrane surface, and then the peptide binds to the membrane on the bacterial surface, which in turn leads to the displacement of the phospholipid membrane to form a cleft, and the insertion of nano-AMPs causes bacterial lysis and death [117].

**Figure 9:**
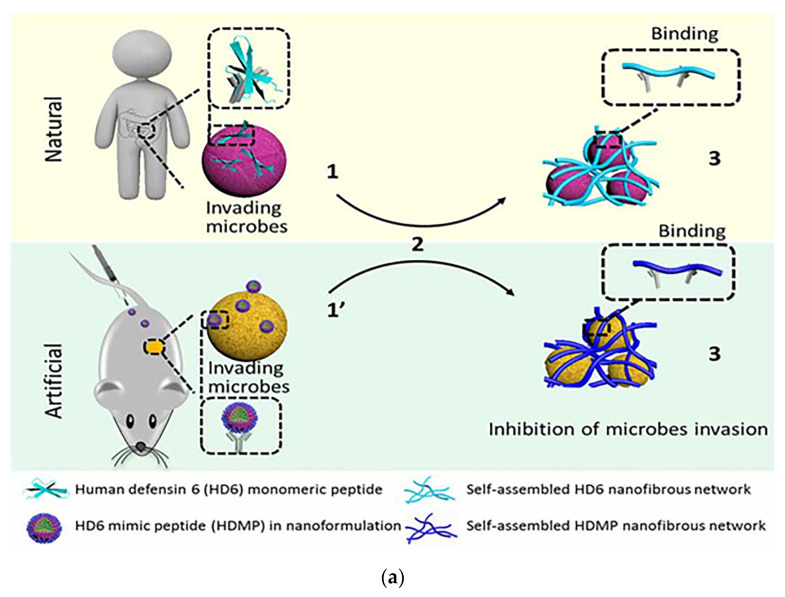

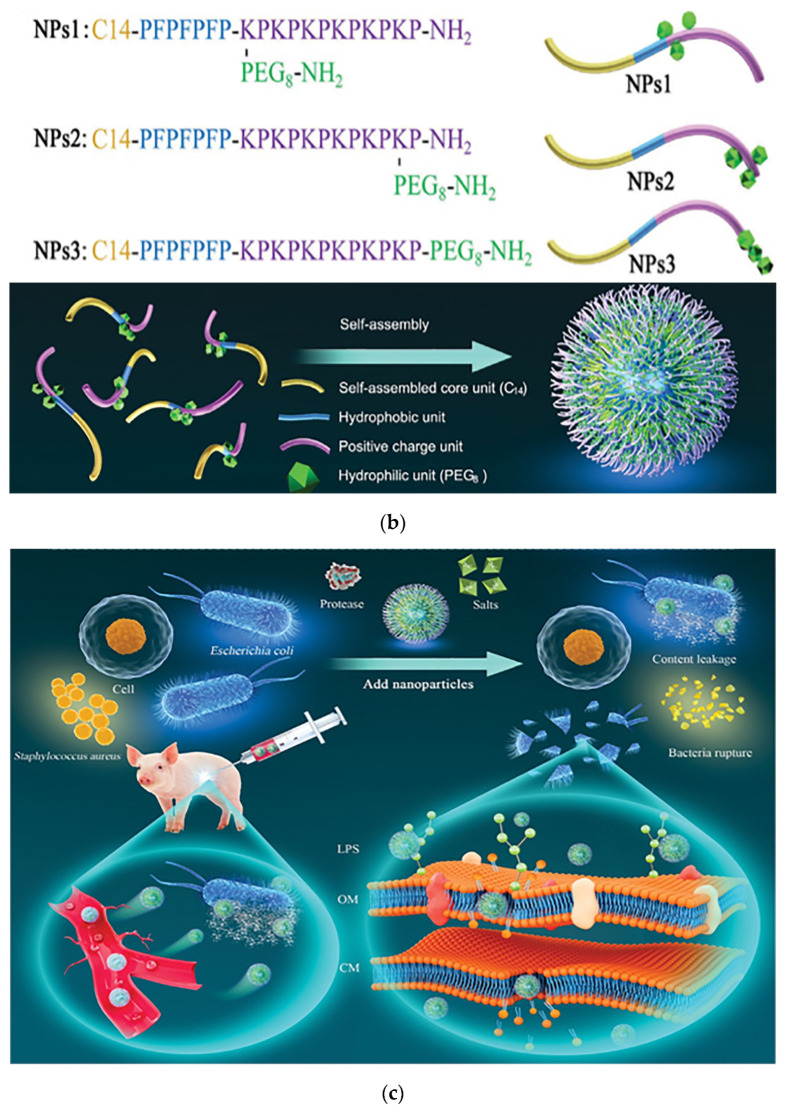

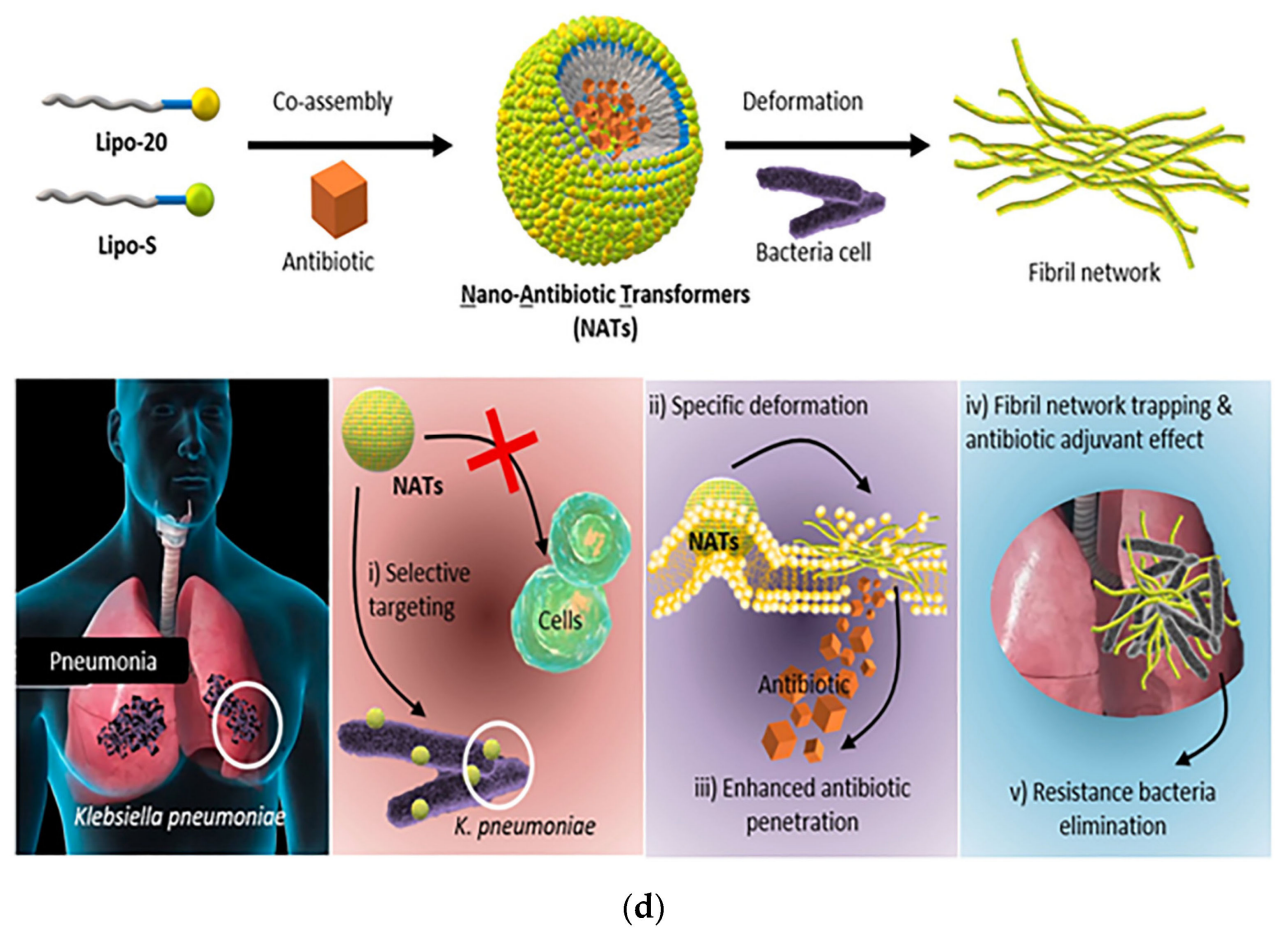
(**a**) Schematic of the mechanism of antibacterial action of bio-nanoparticles that self-assembled to form nanofibers to surround bacteria. The HDMP mimics the HD6 that (2) in situ self-assembly on the microbial surface through ligand-receptor interactions to form (3) a self-assembled trapping fiber network to inhibit microbial invasion. Unlike HD6, which is (1) secreted in situ from Paneth cells, HDMP is (1′) delivered to the target region in the form of nanoparticles (NP). Adapted with permission from [118], Copyright 2020 American Association for the Advancement of Science. (**b**) Schematic representation of the molecular structure and self-assembly process of peptide nanoparticles. (**c**) Schematic of the in vivo sterilization mode of peptide self-assembled nanoparticles. LPS: lipopolysaccharide; OM: outer membrane; CM: cytoplasmic membrane. Adapted with permission from [119], Copyright 2022 John Wiley and Sons. (**d**) The principle of co-assembly of NATs and bacterially induced morphological transformation. Adapted with permission from [120], Copyright 2022 Elsevier.

Fan et al. mimicked the human defensin-6 design with a human defensin-6 mimic peptide (HDMP). The HDMP bis-pyrene-KLVFF-RLYLRIGRR consists of three fragments. The sequence RLYLRIGRR targets the recognition and binding of lipophosphatidic acid (LTA) of Gram-positive bacteria. The self-assembling sequence KLVFF is the peptide backbone that accomplishes molecular assembly. HDMP spontaneously formed self-assembled nanoparticle (NP) formulations that underwent a morphological transformation into nanofibers (NFs) upon binding to the LTA of *S. aureus* (Figure 9a). The nanofibers efficiently and precisely inhibited bacterial invasion into host cells by capturing bacteria. Experimental results show that intravenous injection of 5 mg/kg HDMP in MRSA-infected mice achieved a 100% survival rate, which was higher than the therapeutic effect achieved by vancomycin injection at the same amount (83.3% survival rate) [118]. Tan et al. used the self-assembly strategy to design self-assembled chimeric NPs consisting of alkyl chains, amino acid sequences and PEG units for the treatment of bacterial infections. In designing the sequence, the researchers not only considered the substitution of amino acids that are susceptible to enzymatic cleavage, but also discussed the insertion position of the hydrophilic polyethylene glycol (PEG) structural domain. The protease stability and biocompatibility of the nanoparticles were further improved by regulating the position of PEG in the peptide chain to prevent non-specific protein adsorption (Figure 9b). NPs1 and NPs2 were shown to possess broad-spectrum antibacterial activity, good biocompatibility, and excellent salt and protease stability. The peptide nanoparticles were injected intraperitoneally into piglets, and the particles, which had excellent stability, killed the bacteria in the piglets through a membrane disruption mechanism (Figure 9c). In vivo experiments demonstrated that NPs1 and NPs2 have low toxicity and alleviated systemic bacterial infections in mice and pigs. Unlike conventional antibiotics, the specific membrane permeation mechanism and interference with the cell cycle make the development of resistance difficult [119].

Recently, researchers have begun to construct in situ self-assembled peptide materials that respond to specific stimuli in living cells as well as in animals. The fine-tuning of peptide structures and assembly strategies enables the diagnosis and treatment of a variety of major diseases such as antimicrobial infections. For example, Wang’s team used an in situ self-assembly strategy to design an antimicrobial material with specific targeting and aggregation-induced retention effects. They designed a chitosan–peptide conjugate (CPC) as an enzyme-responsive fragment, which self-assembled into NPs that were cleaved when encountering gelatinase secreted around bacteria, thereby facilitating self-assembly and reorganization into NFs. The NFs were trapped within the infected tissue, leading to accumulation and long-term retention of the nanomaterials, and thus they exhibited an effective antimicrobial capacity [121].

Self-assembled peptide DDSs typically carry drugs by physical encapsulation or chemical coupling, and drugs are released by the breakdown of nanostructures or the breaking of coupling bonds [122]. Based on the concept of an antibiotic adjuvant, Liu et al. successfully designed co-assembled lipopeptides containing antibiotics and nano-antibiotic transformers (NATs) capable of delivering ciprofloxacin, for targeted delivery of antibiotics with the help of nano-assemblies. The first two individual molecules (Lipo-20 and Lipo-S) were designed to complete self-assembly by adjusting the mixing ratio of the two to form nanoparticles. Simultaneous mixing with antibiotics encapsulated the antibiotics in the nanoparticles. The co-assembled lipopeptide was encapsulated with antibiotics that specifically recognized *K. pneumoniae* and underwent deformation, transforming from nanoparticles to nanofibers (Figure 9d). The bactericidal mechanism of NATs followed three steps: (1) NATs specifically recognized *K. pneumoniae*; (2) NATs underwent a specific morphological transformation when encountering exposed bacterial membranes; and (3) conformational transformation by NATs in situ enhanced the permeability of the encapsulated antibiotic. The dual antibacterial effect of bacterial trapping by the nanofiber network and antibiotics released by the bacteria in situ was the winning strategy of the system against drug-resistant bacteria. The optimized ratio of the two components of the lipopeptide maintained high antibiotic adjuvant activity and significantly reduced hemolysis, and it lowered the minimum antibiotic inhibitory concentration by 8-fold. The Lipo-20 peptide acted as a targeting peptide that specifically bound to bacterial membranes and activated the in situ transformation of the nanoparticle structure of the nanoassemblies into nanofibers, thereby enhancing membrane disruption and antibiotic penetration. The delivery system was able to achieve a drug delivery efficiency of over 70% and also significantly enhanced antibiotic longevity with no resistance developing in bacterial dosing experiments over one month [120]. Therefore, antibiotic delivery systems based on peptide self-assembly are a promising strategy for combating bacterial resistance.

## 5. Concluding Remarks and Outlook

Antibiotics are a double-edged sword. The development and application of antibiotics have been described as “one of the greatest scientific and technological achievements of the 20th century”. A large number of antibiotics with various bactericidal mechanisms have given humans a powerful weapon to fight against death, saving the lives of millions of patients with bacterial infections. Antibiotics have been an irreplaceable anti-infection drug up to the present day. However, the overuse of antibiotics has exacerbated the development of bacterial resistance, which also puts humans at increasing risk. The various types of antibiotics and antibacterial mechanisms lead to complex mechanisms of resistance, especially the emergence of MDR bacteria, which brings great challenges to the field. This makes it necessary to explore and research the bactericidal mechanism of antibiotics and the mechanism of bacterial resistance. In addition, the rise of materials science and pharmacy has offered great opportunities for research. Polymer materials, antibacterial peptides and DDS technology have great potential for overcoming bacterial resistance in the future.

At present, most research has focused on finding alternatives to antibiotics. Antimicrobial materials are relatively simple, but there are problems that cannot be ignored, such as the metabolism and toxicity problems of metal nanomaterials. Moreover, the clearance of antimicrobial materials in vivo is always characteristically rapid. Accordingly, DDSs have been developed with many advantages due to their nanoscale advantages, including long in vivo circulation, reduced drug toxicity and increased drug-specific targeting, resulting in improved efficacy. The greatest bottleneck is the biosafety of the carriers, which is also the greatest obstacle to their entry into clinical research. Therefore, much research work is needed to industrialize and extend their application potential. However, the fact that peptides are now available on the market reinforces our belief that novel technologies and scientific findings will be introduced into the clinic for practical antibacterial use in the future.

## Figures and Tables

**Table 1:** Classification of traditional antibiotics.

Name	Representative Drugs	Description	Refs.
β-lactam antibiotics	Penicillin, Cephalosporin, Carbapenem, Monobactams	Widely used in clinical treatment, mainly for infectious diseases caused by sensitive bacteria.	[14,15]
Glycopeptides	Vancomycin	Clinical “last resort” for the treatment of serious infections caused by Gram-positive bacteria.	[16]
Lipopeptides	Daptomycin	A highly successful intravenously injectable natural lipopeptide antibiotic whose antibiotic properties make it a key drug in the treatment of drug-resistant Gram-positive infections.	[17]
Aminoglycosides	Streptomycin, Gentamycin	They have excellent activity and low bacterial resistance in the treatment of infections but are hampered by dose-dependent toxic effects in patients with nephrotoxicity or ototoxicity.	[18]
Tetracyclines	Streptomycin, Minocycline, Doxycycline	Broad-spectrum antibiotics used in the treatment of bacterial infections, periodontitis and skin diseases.	[19]
Macrolides	Erythromycin, Clarithromycin, Azithromycin	Play a key role in the treatment of respiratory tract infections.	[20]
Oxazolidinones	Linezolid	The first FDA-approved oxazolidinone antibiotic, a broad-spectrum Gram-positive drug.	[21]
Quinolones	Norfloxacin, Ofloxacin, Ciprofloxacin, Flurofloxacin	The most used and effective clinical application is the treatment of genitourinary tract infections; they are also widely used in the respiratory system, as well as the intestinal system.	[22]
Ansamycins	Geldanamycin, Herbimycin	This class of antibiotics has a variety of biological activities such as antibacterial, antitumor and antiviral. Some of them are currently clinically important anti-tuberculosis drugs.	[23]
Sulfonamides	Mafenide, Sulfacetamide, Sulfamethizole	Clinical antibiotics of the sulfonamide class can be used for infections in several sites, including the respiratory system, urinary system and several other sites.	[24]
Chlorampheicols	Chloromycetin, Thiamphenicol	They are commonly used clinically for the treatment of infections caused by various sensitive bacteria.	[25]
Lincosamides	Lincomycin, Clindamycin	Lincosamides are a clinically important family of antibiotics, and its representative member, lincomycin, has long been used to treat infections caused by Gram-positive bacteria.	[26]
Furan antibiotics	Furazolidone, Furantoin, Furacilin	They can be used to treat dysentery, enteritis, gastric ulcers and other gastrointestinal disorders caused by bacteria and protozoa.	[27]

**Table 2:** Applications of antibiotic liposomes in the antibacterial field.

Name	Applications	Ref.
Liposomal Amikacin	Mycobacterium avium complex lung disease	[97]
Liposomal Clarithromycin	Clinical isolates of *P. aeruginosa* from the lungs of cystic fibrosis patients	[98]
Liposomal polymyxin B	Higher drug penetration into pulmonary epithelial lining fluid, which resulted in superior efficacy	[99]
Liposomal amphotericin B	An excellent candidate for the first-line treatment of patients with suspected fungal infection, based on prolonged neutropenic fever not responding to antibacterial therapy	[100]
Liposomal Mupirocin	Therapeutic potential for infections involving MDR bacteria	[101]
Liposomal Vancomycin	Applied directly on MRSA-infected skin wounds in mice	[102]
Liposomal Daptomycin and Clarithromycin	Treatment of MRSA infection	[103]

## Data Availability

Not applicable.
